# Multi-Feature Dynamic Reconstruction of Photovoltaic Systems with Battery Storage for Real-Time Grid Monitoring

**DOI:** 10.3390/s26144633

**Published:** 2026-07-22

**Authors:** Tao Xia, Mingqi Lu, Ziyan Ding, Haitao Liu, Lei Huang

**Affiliations:** 1 School of Electric Power Engineering, Nanjing Institute of Technology, Nanjing 211167, China; t.xia@njit.edu.cn (T.X.); y00450240523@njit.edu.cn (M.L.); y00450230547@njit.edu.cn (Z.D.); 2 School of Electrical Engineering, Southeast University, Nanjing 210096, China; huanglei@seu.edu.cn; 3 Ocean New Energy Engineering Center, Advanced Ocean Institute of Southeast University, Nantong 226010, China

**Keywords:** PV systems with battery storage, multi-feature dynamic reconstruction, real-time grid monitoring, controller hardware-in-the-loop

## Abstract

Real-time grid monitoring of photovoltaic (PV) systems with battery storage requires continuous access to voltage, current, and power states. Traditional simulation models obtain these responses by calculating switching events, which limits the scale of real-time simulation. This paper proposes a Multi-Feature Dynamic Reconstruction (MFDR) method that combines environmental inputs, averaged converter states, and frequency-domain electrical variables. On the DC side, PV output is calculated from irradiance and temperature, while the bidirectional battery converter is represented by a low-frequency reconstruction model. On the AC side, dynamic phasors are used to convert the grid-connected inverter into a frequency-domain Norton equivalent for reconstructing its port voltage and current responses. Controller hardware-in-the-loop tests are conducted under different operating conditions. The reported peak and normalized tracking errors of the evaluated transient quantities remain below 3%. In the four-core IEEE 118-bus case, the average per-core CPU utilization decreases from 70.43% to 41.08%, while the maximum step execution time decreases from 38 μs to 27 μs. The model with 1069 state variables also operates within the fixed 50 μs simulation step. The results show that the MFDR method reduces the computational demand of real-time grid monitoring while retaining the voltage, current, and power responses.

## 1. Introduction

The increasing scale of grid-connected photovoltaic (PV) plants has created a growing demand for continuous monitoring of voltage, current, power, and operating status [1]. By the end of 2025, China’s installed renewable-energy capacity reached 2.34 TW, an increase of 24% year on year, including 1.20 TW of solar power capacity [2]. This expansion is increasing both the share of renewable generation and the number of power-electronic interfaces in the grid. Electromagnetic transient (EMT) simulation is widely used to analyze converter transients, control interactions, and network responses in such systems [3,4,5]. However, as more converter-interfaced units are connected, the number of switching states and the size of the time-varying network equations increase rapidly. The resulting computational burden limits the scale of detailed EMT simulation [6].

Currently, detailed EMT models of grid-connected PV systems usually represent the PV array, power converters, controllers, and grid interface through coupled circuit and control equations [7]. When battery storage and its bidirectional converter are included, the number of state variables and switching events increases further. These models can reproduce detailed electrical responses, but their computational cost restricts the number of units that can be simulated in real time. A more efficient model is therefore needed to preserve the voltage, current, and power responses required for grid monitoring.

At the PV-unit level, irradiance and temperature are commonly monitored together with voltage, current, and power because they directly affect the available output [8]. Five-parameter and seven-parameter models can represent the nonlinear current–voltage characteristics of PV modules [9,10]. At the plant level, single-unit equivalent models have been developed for large PV plants, with fault-ride-through behavior corrected to improve transient equivalence [11]. These methods improve PV-module representation and plant-level aggregation, but they do not address the combined real-time modeling of the PV unit, battery converter, and grid-connected inverter.

Several methods have been proposed to reduce computational cost while retaining selected transient characteristics. Adjustable-resolution models use layered decoupling to vary the level of detail according to the simulation task [12]. Co-simulation frameworks combine EMT and frequency-domain calculations through phasor extraction [13]. Small-signal averaged models are suitable for impedance- and admittance-based stability analysis near a selected operating point, but their accuracy decreases when the system undergoes large disturbances [14]. Average-value and current-injection inverter models can also shorten simulation time, although some waveform details are omitted [15]. Dynamic-phasor models provide another option by retaining selected frequency components of converter and network variables [16]. However, these methods focus on different subsystems or modeling objectives and do not provide a coordinated representation of all three power-conversion stages.

Even with these model reductions, the computational cost of EMT simulation still rises with network size and converter count [17]. The main cost comes from repeatedly solving fast controls, time-varying converter states, and switching-dependent network equations. Existing acceleration methods therefore use system equivalents, simplified component models, parallel algorithms, or dedicated hardware [18]. Digital-twin and IoT platforms also require virtual models to update at rates compatible with monitoring and control [19]. Hardware-oriented optimization can shorten the execution time of large renewable-energy models [20], while shifted-frequency modeling and parallelization can extend the real-time capability of power-electronic network simulation [21].

Among the existing reduced-complexity approaches, average-value and current-injection models reduce switching-event calculations by retaining selected converter responses, while dynamic-phasor models represent converter and network variables using a limited set of frequency components. Representative studies have evaluated the accuracy and computational characteristics of these reduced-order inverter models against detailed EMT benchmarks [15,16]. Adjustable-resolution and co-simulation frameworks improve computational efficiency through model-resolution adaptation or the coupling of different calculation domains [12,13]. Hardware-oriented optimization and parallel computation further improve execution efficiency at the computing-platform level [20,21]. These approaches, however, generally focus on individual converters, selected frequency components, or computational implementation, rather than the coordinated representation of the complete PV–battery system.

To address this limitation, this study proposes a Multi-Feature Dynamic Reconstruction (MFDR) method for grid-connected PV systems with battery storage. The method applies environmental feature mapping to the PV unit, averaged-state reconstruction to the bidirectional battery converter, and frequency-domain reconstruction to the grid-connected inverter. By integrating these subsystem representations into a unified model, the proposed method retains the dominant voltage, current, and power responses required for grid monitoring while reducing the computational burden of large-scale real-time simulation.

The main contributions of this study are summarized as follows:

- A coordinated Multi-Feature Dynamic Reconstruction framework is developed for grid-connected PV systems with battery storage. It integrates environmental feature mapping for the PV unit, averaged-state reconstruction for the bidirectional battery converter, and frequency-domain dynamic reconstruction for the grid-connected inverter into a unified real-time simulation model.
- Low-frequency and frequency-domain Norton equivalent models are formulated for the bidirectional battery converter and grid-connected inverter, respectively. These equivalent models avoid the explicit calculation of converter switching events while retaining the dominant DC-bus voltage, converter current, and grid-side power responses required for system-level grid monitoring.
- The proposed MFDR model is comprehensively evaluated through controller hardware-in-the-loop tests and simulations based on the IEEE 33-bus and IEEE 118-bus systems. Its reconstruction accuracy, computational efficiency, and scalability are quantitatively assessed under irradiance variations, load disturbances, and three-phase short-circuit faults.

Section 2 presents the system topology, operating characteristics, and coordinated control strategies of the grid-connected PV system with battery storage. Section 3 develops the MFDR model, including environmental feature mapping for the PV unit, low-frequency reconstruction of the bidirectional battery converter, and frequency-domain dynamic reconstruction of the grid-connected inverter. Section 4 describes the CHIL platform and evaluates the reconstruction accuracy, computational efficiency, and scalability of the proposed model under different operating conditions and network scales. Section 5 summarizes the main conclusions and discusses the applicability, limitations, and future extension of the proposed method.

## 2. Topology and Operating Characteristics of PV Systems with Battery Storage

### 2.1. System Topology

**Figure 1:**
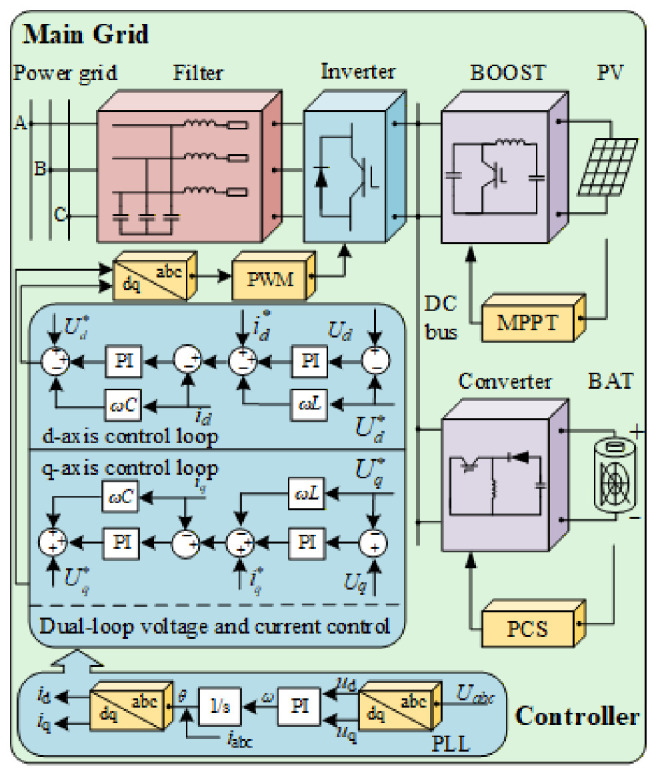
Main circuit and control structure of the PV system with battery storage.

A PV system with battery storage consists of a PV generation unit, a battery energy storage unit, a common DC bus, a grid-connected inverter, and an AC-grid interface. The PV unit is connected to the DC bus through a boost converter, while the battery unit is interfaced through a bidirectional DC–DC converter. The grid-connected inverter regulates the power exchanged between the DC bus and the AC grid. Figure 1 illustrates the system topology and the main control structure. The PV-side boost converter operates with MPPT control to regulate the harvested solar power, whereas the battery-side bidirectional converter controls the charging and discharging process according to the DC-bus power balance. The inverter is responsible for coordinating the DC-bus voltage and the active/reactive power exchange with the utility grid. Through this coordinated structure, the PV unit, battery unit, and inverter jointly determine the system-level voltage, current, and power responses analyzed in the following sections.

#### 2.1.1. Output Characteristics of the PV Module

**Figure 2:**
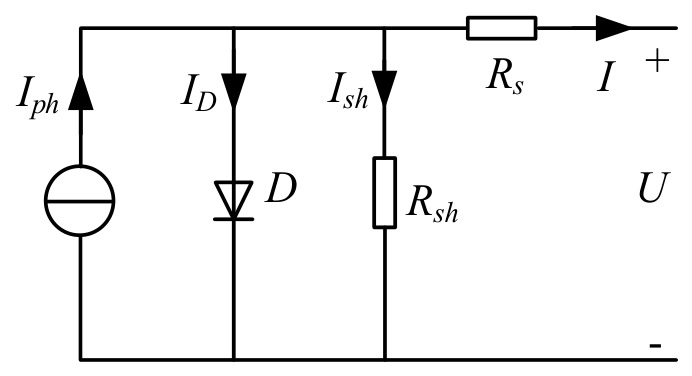
Equivalent single-diode circuit of the PV module.

The PV module is represented by an equivalent single-diode model. Its current–voltage characteristic is nonlinear and depends mainly on irradiance and module temperature. The equivalent circuit adopted in this study is shown in Figure 2.

**Figure 3:**
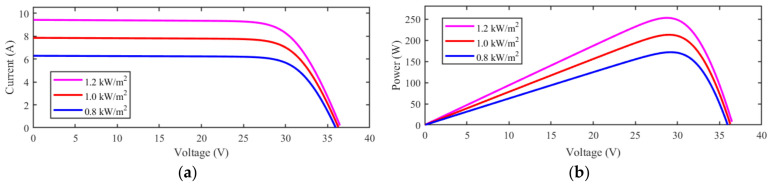
Output characteristics of the PV module at different irradiance levels. (**a**) *I*–*V* characteristics. (**b**) *P*–*V* characteristics.

The output current of the PV module is expressed as follows:

(1) $$I=I_{ph}-I_{D}(e^{\frac{q}{n_{id}kT}(U+IR_{s})}-1)-\frac{1}{R_{sh}}(U+IR_{s})$$

where *I* and *U* denote the PV-module output current and voltage; *I_ph_* and *I_D_* are the photocurrent and diode reverse saturation current; *q* = 1.6 × 10^−19^ C is the elementary charge; *n_id_* is the diode ideality factor; *T* is the temperature; *k* = 1.38 × 10^−23^ J/K is the Boltzmann constant; and *R_s_* and *R_sh_* are the series and shunt resistances. Figure 3 shows the output characteristics of a single PV at 25 °C and irradiance levels of 0.8, 1.0, and 1.2 kW/m^2^.

Figure 3a,b show that the output current of the PV module increases with irradiance, and the output voltage changes within a narrower range. The maximum power point shifts as the irradiance varies. The MPPT controller therefore adjusts the duty cycle of the boost converter so that the PV operating point tracks the maximum power point.

#### 2.1.2. Output Characteristics of the Battery Energy Storage Unit

**Figure 4:**
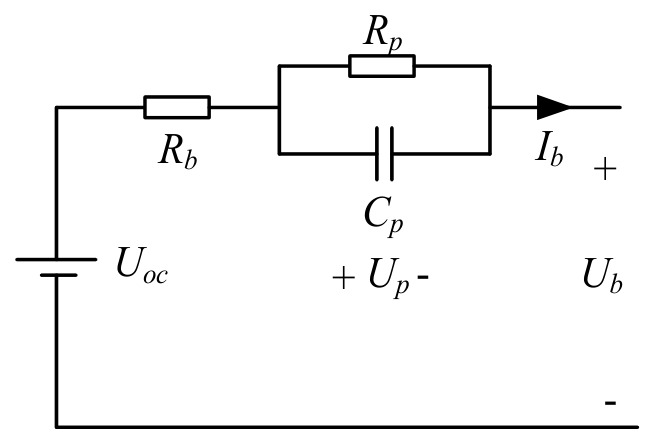
Equivalent circuit of battery energy storage unit.

The battery energy storage unit compensates for short-term differences among PV generation, load demand, and grid-side power exchange. A first-order Thevenin equivalent circuit is used to represent its terminal behavior. As shown in Figure 4, the ideal voltage source *U_oc_* represents the open-circuit voltage of the battery, which is related to the state of charge (SOC). The parallel *R_p_C_p_* network represents the polarization dynamics, while *R_b_* represents the battery ohmic resistance.

According to Kirchhoff’s voltage law, the terminal output voltage *U_b_* of the battery is expressed as

(2) $$U_{b}=U_{oc}(SOC)-I_{b}R_{b}-U_{p}$$

where *I_b_* is defined as positive during battery discharge. *U_p_* is the polarization voltage. The polarization dynamics are governed by the time constant *τ_p_* = *R_p_C_p_*. The model describes the terminal-voltage variation caused by the state of charge, ohmic voltage drop, and polarization process. In the present study, *U_oc_*, *R_b_*, *R_p_*, and *C_p_* are treated as fixed parameters over each short-duration test. The model is therefore intended for limited SOC excursions and approximately isothermal conditions around the parameterized operating point. SOC- and temperature-dependent parameter variations, voltage hysteresis, and aging-induced impedance changes are not represented.

### 2.2. System Control Strategies and Operating Modes

The operation of the PV system with battery storage depends on the coordinated control of its power conversion stages. As shown in Figure 1, the overall control architecture of the system uses the DC bus as the hub for power exchange. The control system consists of coordinated PV and battery storage control on the DC side and inverter control on the AC side. Together, these control layers regulate the DC-bus power balance and the power exchanged with the grid.

#### 2.2.1. Coordinated PV and Battery Storage Control on the DC Side

The DC-side controller extracts the available PV power and regulates the power balance of the common DC bus. The MPPT module measures the PV-side voltage and current, calculates the instantaneous power using the perturb-and-observe (P&O) method, and updates the duty cycle of the boost converter. The PV operating point therefore tracks the maximum power point of the *P*–*V* curve as irradiance and temperature vary.

The battery storage system is connected to the DC bus through a bidirectional power conversion system (PCS). When the PV power exceeds the load and grid-side demand, the converter operates in buck mode and charges the battery. Conversely, the converter operates in boost mode and discharges the battery. This operation reduces the instantaneous power imbalance at the DC bus.

#### 2.2.2. Grid-Connected Inverter Control on the AC Side

The grid-connected inverter adopts cascaded DC-link-voltage and current control in the synchronous *dq* reference frame. A phase-locked loop (PLL) estimates the grid-voltage phase angle and frequency. The three-phase voltages and currents are then transformed into the corresponding *dq*-axis components.

The outer voltage loop regulates the DC-link voltage and generates the *d*-axis current reference. The *q*-axis current reference is determined by the reactive-power command. The inner current loop employs grid-voltage feedforward and cross-coupling compensation to regulate the *d*- and *q*-axis currents independently.

## 3. Multi-Feature Dynamic Reconstruction Model for PV Systems with Battery Storage

To reduce the computational burden of real-time simulation while retaining the main electrical responses required for grid monitoring, this section develops a Multi-Feature Dynamic Reconstruction (MFDR) model for PV systems with battery storage. The model reconstructs the PV unit, battery converter, and grid-connected inverter using their environmental inputs, averaged state variables, and frequency-domain electrical variables, respectively. These physically defined features provide the basis for the reconstruction methods presented in the following sections.

### 3.1. PV-Side Reconstruction Based on Environmental Feature Mapping

To reduce the computational cost of the traditional PV model, the PV module and its boost converter are represented by a controlled source driven by irradiance and temperature. It directly establishes the mapping relationship between the environmental input variables, irradiance *S* and temperature *T*, and the DC-side maximum output power *P_m_*.

Under Standard Test Conditions (STC), the reference irradiance and temperature are *S_ref_* = 1000 W/m^2^ and *T_ref_* = 25 °C respectively. Under these conditions, the short-circuit current *I_sc___stc_* and open-circuit voltage *U_oc___stc_*, as well as the current *I_m___stc_* and voltage *U_m___stc_* at the maximum power point, can be obtained from manufacturer data.

Because the PV current varies approximately linearly with irradiance, the short-circuit current and maximum-power-point current under arbitrary operating conditions are calculated as follows:

(3) $$I_{sc}=I_{sc\text{\_}stc}\left[1+a(T-T_{ref})\right]\cdot \frac{S}{S_{ref}}$$

(4) $$I_{m}=I_{m\text{\_}stc}\left[1+a(T-T_{ref})\right]\cdot \frac{S}{S_{ref}}$$

where *a* is the current temperature coefficient. For the PV module considered in this study, a = 0.0025/°C is adopted from the published PV parameter-correction formulation in [22], rather than being re-identified for the selected module.

Unlike the current characteristics, the output voltage of the PV module is more sensitive to temperature changes and exhibits nonlinear characteristics with irradiance variations. Therefore, a logarithmic term is used to represent the irradiance dependence of the open-circuit voltage and maximum-power-point voltage:

(5) $$U_{oc}=U_{oc\text{\_}stc}\ln[e+b(S-S_{ref})]\left[1-c(T-T_{ref})\right]$$

(6) $$U_{m}=U_{m\text{\_}stc}\ln[e+b(S-S_{ref})]\left[1-c(T-T_{ref})\right]$$

where *b* is the irradiance correction coefficient and *c* is the voltage temperature coefficient. The values *b* = 0.0005 and *c* = 0.00288/°C are adopted from the published parameter-correction formulation in [22]. The standard-condition electrical parameters *Isc_stc*, *Uoc_stc*, *Im_stc*, and *Um_stc* remain module-specific.

The available maximum power under the specified irradiance and module temperature is calculated from the corrected MPP voltage and current:

(7) $$P_{m}=U_{m}\cdot I_{m}=U_{m\text{\_}stc}\cdot I_{m\text{\_}stc}\cdot \frac{S}{S_{ref}}\cdot (1+a\Delta T)\ln(e+b\Delta S)(1-c\Delta T)$$

where Δ*T* = *T* − *T_ref_* and Δ*S* = *S* − *S_ref_*. Based on the principle of power conservation, the equivalent current command *I_ref_* injected into the DC bus can be expressed as

(8) $$I_{ref}=\frac{P_{m}}{U_{dc}}$$

where *U_dc_* is the sampled value of the DC-bus voltage. The coefficients used in Equations (3)–(6) are empirical values reported in the literature and were not re-fitted using module-specific *I*–*V* measurements in this study. Consequently, no module-specific fitting residual is reported. For applications requiring higher parameter accuracy, the coefficients should be recalibrated using manufacturer data under multiple irradiance and temperature conditions or identified from measured *I*–*V* characteristics.

**Figure 5:**
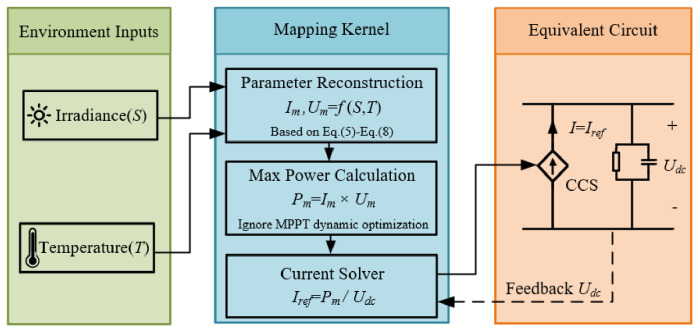
Environmental feature-mapping architecture for PV-side reconstruction.

Based on the parameter correction model described above, this paper constructs a three-level mapping modeling architecture as shown in Figure 5. This architecture organizes the PV-side reconstruction into three layers: The environmental input layer introduces irradiance *S* and temperature *T* as input variables. The feature-mapping layer serves as the computational core of the model. It reconstructs the *I*–*V* characteristic parameters under the current operating conditions and calculates the maximum available power. The DC-bus voltage feedback *U*_dc_ is then used to obtain the equivalent current command *I_ref_*, thereby linking the bus voltage to the injected current. The interface equivalent layer uses a controlled-current source (CCS) to replace the PV module and the boost converter. This mapping converts the switching-dependent differential calculation into an algebraic source calculation and retains the PV-side power response required by the system-level model.

### 3.2. Battery Converter Reconstruction Using a Low-Frequency Reconstruction Model

The battery is connected to the common DC bus through a bidirectional DC–DC converter. The high-frequency switching actions of its power devices introduce significant nonlinear characteristics and increase the computational load of real-time simulation. In the MFDR model, the inductor current and DC-bus voltage are treated as averaged state features. A low-frequency reconstruction model (LFRM) is then used to replace the switching converter with an equivalent controlled-source interface while retaining its dominant state evolution.

**Figure 6:**
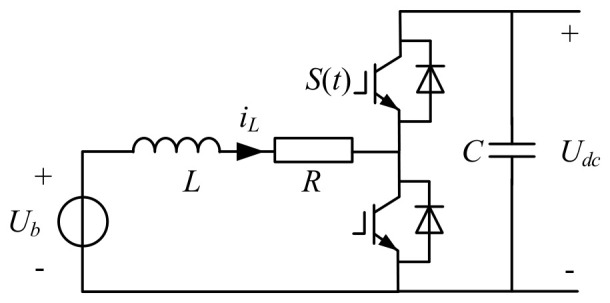
Bidirectional battery converter circuit topology.

The bidirectional DC-DC converter adopts a bidirectional half-bridge topology, as shown in Figure 6. It consists of an inductor *L*, a DC-bus capacitor *C*, and two complementary switches.

To characterize the energy exchange process between the battery side and the bus side, the energy storage inductor current *i_L_* and the DC-bus voltage *u_dc_* are selected as state variables. Following the established state-space averaging approach for bidirectional DC–DC converters [23], these variables are used to construct the state vector ***x***(***t***) = [*i_L_*(*t*), *u_dc_*(*t*)]*^T^*. According to Kirchhoff’s voltage and current laws, the converter is described by the following switching state equation:

(9) $$\frac{dx(t)}{dt}=A_{0}x(t)+b_{0}+[(A_{1}x(t)+b_{1})S(t)]$$

where *S*(*t*) ∈ {0,1} is the switching function of the upper switch; 1 denotes on-state and 0 denotes off-state. Matrices ***A*_0_** and ***b*_0_** represent the parameter matrices in the freewheeling mode. When *S*(*t*) = 0, the inductor *L* is disconnected from the DC bus and the bus capacitor supplies the connected load:

(10) $$A_{0}=\left[\begin{array}{cc} -R/L & 0\\ 0 & 0 \end{array}\right],b_{0}=\left[\begin{array}{c} U_{bat}/L\\ -I_{L}/C \end{array}\right]$$

***A*_1_** and ***b*_1_** describe the coupled mode. When *S*(*t*) = 1, the inductor *L* is connected to the DC bus and transfers energy between the battery and the bus.

(11) $$A_{1}=\left[\begin{array}{cc} 0 & -1/L\\ 1/C & 0 \end{array}\right],b_{1}=0$$

Considering that *S*(*t*) is discontinuous, directly solving Equation (9) requires an extremely small simulation step to resolve individual switching transitions. The period-average switching state *S_avg_*(*t*) is therefore defined over the switching period *T_s_*, and represented by the duty ratio *d*(*t*). Its physical meaning is the effective conduction density of the power device per unit period:

(12) $$d(t)=S_{avg}(t)=\frac{\int_{t-T_{s}}^{t}S(\tau )d\tau }{T_{s}}$$

where *S*(*τ*) represents the instantaneous value of the switching function within the integration interval, and *τ* denotes the integration time axis within the time window [*t* − *T_s_*, *t*].

**Figure 7:**
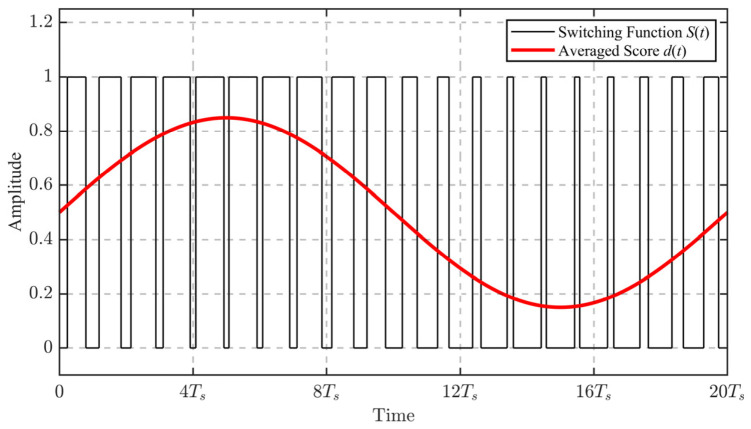
Switching function *S*(*t*) and its periodic average duty cycle *d*(*t*).

As shown in Figure 7 below, the discontinuous switching function is replaced by the continuous duty ratio *d*(*t*). Under the small-ripple assumption, the variation in the state vector within one switching period is small relative to its averaged component. Equation (9) can be reconstructed as the following continuous averaged state equation:

To obtain an algebraic port model for real-time implementation, Equation (13) is discretized using the implicit trapezoidal rule with simulation step Δ*t*.

(13) $$\frac{d\bar{x}(t)}{dt}=A_{0}\bar{x}(t)+A_{1}\bar{x}(t)d(t)+b_{0}$$

In EMT-type simulation, this discretization converts dynamic elements into Norton companion representations consisting of an equivalent conductance and a history-dependent source [24]:

(14) $$M(d_{t})\cdot \bar{x}(t)=N_{t-\Delta t}\cdot \bar{x}(t-\Delta t)+\frac{\Delta t}{2}[b_{0}(t)+b_{0}(t-\Delta t)]$$

The discretization coefficient matrices ***M***(***d_t_***) and ***N_t_*** are defined as

(15) $$\left\{\begin{array}{c} M(d_{t})=E-\frac{\Delta t}{2}A(d_{t})\\ N(d_{t-\Delta t})=E+\frac{\Delta t}{2}A(d_{t-\Delta t}) \end{array}\right.$$

where ***E*** is the identity matrix, and ***A***(***d***) = ***A*_0_** + ***d***(***t***)***A*_1_**. Extracting the port voltage and current from the discrete state equation gives

(16) $$I(t)=G_{eq}(d_{t})\cdot u_{dc}(t)+i_{L}(t-\Delta t)$$

where ***G_eq_*** is derived from the discretization matrix ***M***(***d_t_***). The state-space averaging and trapezoidal companion discretization employed above follow established principles of converter modeling and EMT simulation. This paper integrates these principles into the LFRM and the overall MFDR framework to construct a time-varying Norton interface for the bidirectional battery converter in the coordinated PV–battery system.

**Figure 8:**
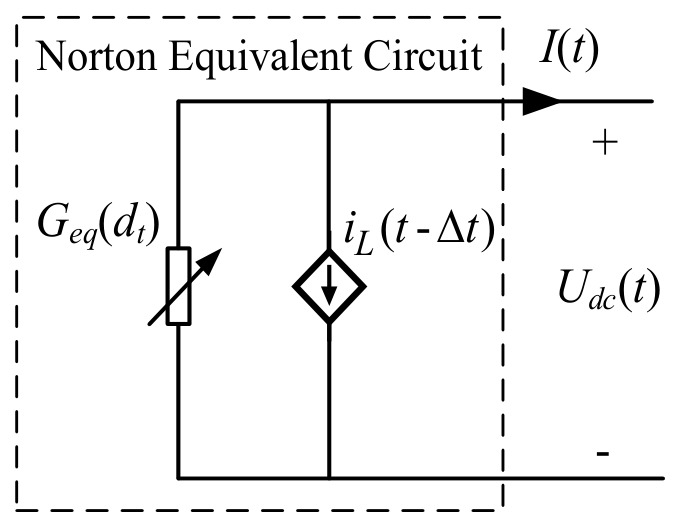
LFRM-based Norton equivalent model for bidirectional battery converter.

Through LFRM, the battery converter is represented by a time-varying conductance in parallel with a history-dependent current source, as shown in Figure 8.

This representation replaces the switching pulses with averaged state features and retains the dominant inductor-current and DC-bus voltage dynamics required by the system-level model. The LFRM reconstructs the averaged converter dynamics, whereas the battery terminal behavior is determined by the first-order Thevenin model. The intended operating envelope is therefore limited to short-duration transient studies around a parameterized SOC and temperature, with the cell condition adequately represented by the adopted battery parameters. For aged or second-life cells, fixed parameters may not capture nonlinear impedance growth, hysteresis, or temperature-dependent behavior, and the reconstruction accuracy may consequently decrease. Such applications require parameter re-identification for the actual battery condition or an SOC-, temperature-, and aging-dependent higher-order battery model.

### 3.3. Grid Inverter Reconstruction Based on Frequency-Domain Dynamic Reconstruction

**Figure 9:**
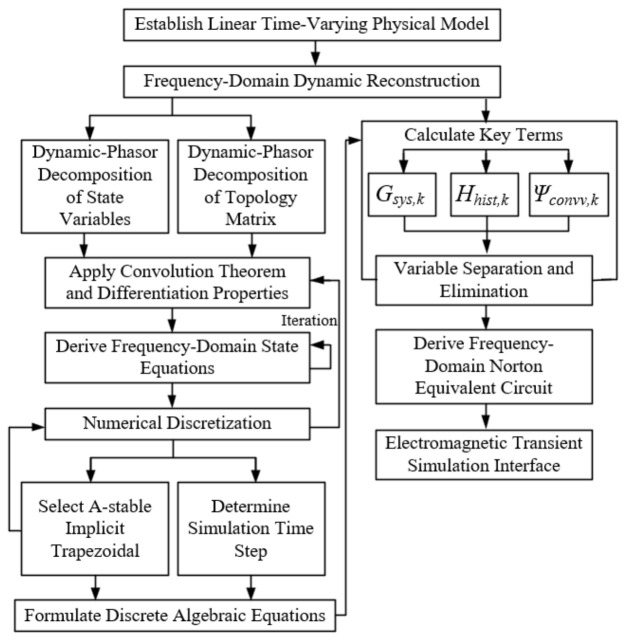
Modeling procedure of the FDDR method for the grid-connected inverter.

The grid-side inverter serves as the energy exchange interface between the DC bus and the AC grid. Unlike the DC characteristic in energy storage converters, its switching states periodically change the coupling among the DC-side voltage, AC-side currents, and grid voltage. Resolving switching events requires a small simulation step. To improve the system simulation efficiency, this section proposes a Frequency-Domain Dynamic Reconstruction (FDDR) method. The FDDR method projects the time-varying state equations into dynamic-phasor coordinates and derives a Norton equivalent interface for the inverter. The modeling procedure is shown in Figure 9:

For the three-phase full-bridge voltage-source inverter, the state vector ***y***(***t***) contains the three filter-inductor currents ***i_abc_***(***t***) = [*i_a_*(*t*), *i_b_*(*t*), *i_c_*(*t*)]*^T^* and the DC-bus voltage *u_dc_*(*t*). The input vector ***u***(***t***) = [*u_abc_*, *i_dc_*]*^T^* is composed of the three-phase grid voltage ***u_abc_***(***t***) = [*u_a_*(*t*), *u_b_*(*t*), *u_c_*(*t*)]*^T^* and the DC-side injected current *i_dc_*.

Unlike the characteristics in energy storage converters, the inverter switching states directly modify the topology matrix of the circuit. Its instantaneous behavior is therefore represented by the following linear time-varying state equation:

(17) $$M\frac{dy(t)}{dt}=\left[A+B(t)\right]y(t)+Cu(t)$$

where ***M*** is the energy-storage coefficient matrix formed by the AC-side filter inductance *L* and the DC-side support capacitance *C*, and is a diagonal positive definite matrix:

(18) M=diag(L,L,L,C)

***A*** is the constant system matrix, containing the resistive and passive circuit terms that are independent of the switching state.

(19) A=diag(−R,−R,−R,0)

***B***(***t***) is the time-varying topology mapping matrix, whose elements consist of the three-phase switching functions *S_k_*(*t*) where *k* ∈ {*a*, *b*, *c*}:

(20) $$B(t)=\left[\begin{array}{cccc} 0 & 0 & 0 & S_{a}(t)\\ 0 & 0 & 0 & S_{b}(t)\\ 0 & 0 & 0 & S_{c}(t)\\ -S_{a}(t) & -S_{b}(t) & -S_{c}(t) & 0 \end{array}\right]$$

***C*** is the input matrix that maps the external voltage and current sources to the state equation.

(21) C=diag(−1,−1,−1,1)

As shown in Equation (17), the computational difficulty arises from the multiplicative coupling between the time-varying topology matrix ***B***(***t***) and the state vector ***y***(***t***). Because this coupling cannot be represented by a single constant system matrix, the state equation is transformed into dynamic-phasor coordinates.

For a time-varying signal *f*(*t*), the *k*-th dynamic phasor is defined over the moving window *T*_0_ as

(22) $${\langle f\rangle }_{k}(t)=\frac{1}{T_{0}}\int_{t-T_{0}}^{t}f(\tau )e^{-jk\omega_{0}\tau }d\tau$$

where *T*_0_ is the fundamental period and *ω*_0_ = 2*π*/*T*_0_.

Applying this operator to the time-varying topology matrix ***B***(***t***) in Equation (17) yields its *l*-th dynamic-phasor coefficient matrix, which is obtained from the Fourier coefficients of the switching functions.

(23) $${\langle B\rangle }_{l}=F_{l}[B(t)]=\left[\begin{array}{cc} 0_{3\times 3} & {\langle S\rangle }_{l}\\ -{\langle S\rangle }_{l}^{T} & 0 \end{array}\right]$$

where ${\left\langle S\right\rangle }_{l}$ is the *l*-th dynamic-phasor coefficient of the switching-function vector.

According to the convolution theorem, the product of two time-varying variables in the time domain corresponds to the discrete convolution of their frequency-domain coefficients. Therefore, the projection of the bilinear term ***B***(***t***)***y***(***t***) in the *k*-th dynamic-phasor subspace can be expressed as a convolution sum of the matrix sequences:

(24) $$F_{k}[B(t)y(t)]=\sum_{l=-K}^{K}{\langle B\rangle }_{l}\cdot {\langle y\rangle }_{k-l}$$

where *K* is the maximum retained dynamic-phasor order, resulting in 2*K* + 1 subspaces. Combining the differential properties of dynamic phasors gives

(25) $$F_{k}[\frac{dx}{dt}]=\frac{d}{dt}{\langle y\rangle }_{k}+jk\omega_{0}{\langle y\rangle }_{k}$$

Applying the *k*-th dynamic-phasor projection to Equation (17) gives the frequency-domain state equation,

(26) $$M\frac{d{\langle y\rangle }_{k}}{dt}=L_{k}{\langle y\rangle }_{k}+\sum_{l=-K}^{K}{\langle B\rangle }_{l}{\langle y\rangle }_{k-l}+C{\langle u\rangle }_{k}$$

where $L_{k}$ = ***A*** − *jkw*_0_***M***, and *jkw*_0_***M*** is the frequency rotation factor associated with the *k*-th dynamic-phasor component. To achieve the numerical solution of the frequency-domain differential Equation (26) in EMT programs, it must be transformed into a port circuit model in algebraic form. The implicit trapezoidal integration method with A-stable characteristics is adopted to replace the differential operator in Equation (26) with a difference approximation. Setting the simulation step as Δ*t*, the discretized algebraic equation for the *k*-th order dynamic phasor at time *t* can be obtained:

(27) $$G_{sys,k}\cdot {\langle y\rangle }_{k}(t)=H_{hist,k}(t-\Delta t)+\Psi_{conv,k}(t)$$

where ***G***_*sys*,*k*_ is the system conductance matrix containing the energy-storage coefficient matrix ***M***, constant system matrix ***A***, and frequency rotation factor *jkw*_0_. Since the full system state vector ***y***(***t***) consists of the three-phase inductor currents *i_abc_* and the DC-bus voltage *u_dc_*, its dimension is 4. Consequently, the corresponding system conductance matrix ***G***_*sys*,*k*_ is defined as a 4 × 4 matrix. For a fixed simulation step Δ*t*, this matrix is constant and expressed as

(28) $$G_{sys,k}=M-\frac{\Delta t}{2}\left(A-jk\omega_{s}M\right)$$

***H***_*his*,*k*_ contains the contribution of the state variables from the previous simulation step.

(29) $$H_{hist,k}(t-\Delta t)=\left(M+\frac{\Delta t}{2}L_{k}\right){\langle y\rangle }_{k}(t-\Delta t)$$

***Ψ***_*conv*,*k*_ represents the cross-frequency coupling among the retained dynamic-phasor components introduced by the time-varying topology matrix ***B***(***t***).

(30) $$\Psi_{conv,k}(t)=\sum_{l=-K}^{K}{\langle B\rangle }_{l}(t)\cdot {\langle y\rangle }_{k-l}(t)$$

Based on Equation (27), the AC-port voltage and current are extracted from the state vector to obtain the frequency-domain Norton relation for the *k*-th dynamic-phasor component:

(31) $${\langle i_{ac}\rangle }_{k}(t)=Y_{eq,k}\cdot {\langle u_{ac}\rangle }_{k}(t)+J_{eq,k}(t)$$

where ***Y***_*eq*,*k*_ is the equivalent admittance matrix relating the AC-port voltage and current, and ***J***_*eq*,*k*_ is the equivalent parallel current source. The DC-bus state remains included through the inverse of ***G***_*sys*,*k*_.

**Figure 10:**
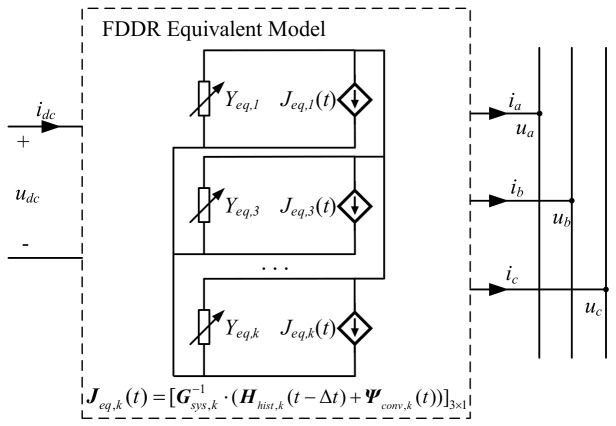
Frequency-domain Norton equivalent of the grid-connected inverter based on FDDR.

The resulting frequency-domain Norton equivalent is shown in Figure 10.

In the equivalent network, each retained dynamic-phasor component is represented by a Norton branch connected to the AC interface. The equivalent current source is determined by the historical-state term and the cross-frequency coupling term. These terms retain the dependence on the previous state and the coupling between the DC and AC sides of the inverter.

Increasing the retained dynamic-phasor order enlarges the coupled frequency-domain system and increases the computational cost of each simulation step. Previous studies have shown that the retained dynamic-phasor components can be selected according to their relative significance and the accuracy–complexity requirements of the intended application [25]. In this study, *K* = 3 is adopted as an engineering-selected truncation order, corresponding to the seven components, from *k* = −3 to *k* = 3. This setting is used to represent the dominant system-level voltage, current, and power dynamics within the fixed 50 μs real-time simulation step.

The appropriate retained order depends on the monitoring bandwidth and the available computational resources. The relative contribution of each retained order to the overall reconstruction error has not been quantified in the present study; therefore, *K* = 3 should be regarded as an application-dependent setting rather than a universally optimal order. A systematic order-sensitivity analysis will be considered in future work.

The MFDR model is intended for system-level real-time studies focusing on the dominant voltage, current, and power responses, such as those caused by irradiance variations, load disturbances, and grid faults. Its applicability is limited by the averaging assumption and the finite dynamic-phasor order, and therefore depends on whether the relevant dynamics are covered by the retained states and frequency components. The model does not retain individual switching transitions or phenomena outside the represented frequency range. Consequently, a traditional switching-level EMT model remains necessary for studies involving switching ripple, semiconductor-level electrical stress, switching losses, very fast electromagnetic transients, or protection functions that depend on sub-cycle or high-frequency waveform details.

## 4. Case Studies and Performance Evaluation

**Figure 11:**
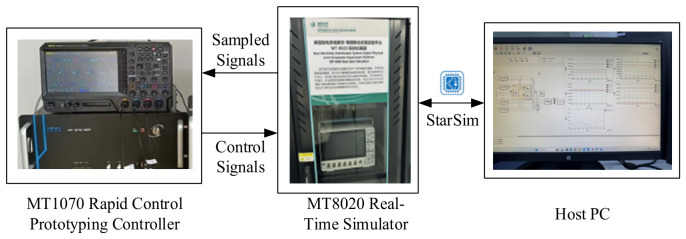
Architecture of the CHIL experimental platform.

A controller hardware-in-the-loop (CHIL) platform was established to assess the reconstruction accuracy and real-time performance of the proposed MFDR model. The system configuration and operating parameters were selected with reference to a grid-connected PV system with battery storage at a production facility in Xiang’an, Xiamen. The traditional switching-based model and the MFDR model were implemented using identical system parameters, control settings, operating conditions, and simulation steps. The traditional model was used as the numerical reference for the waveform and error comparisons presented in this section. As shown in Figure 11, the platform consists of an MT1070 rapid control prototyping controller [26], an MT8020 real-time simulator [27], and a host computer. The MT8020 supports multi-core partitioned execution, allowing different model modules to be assigned to separate CPU cores.

The microgrid test system adopts the PV–battery main-circuit topology and coordinated control architecture shown in Figure 1. Its principal component ratings, load levels, DC-link reference, and simulation step are summarized in Table 1. The detailed conditions of the irradiance-variation, active-load-disturbance, and short-circuit-fault scenarios are provided in Section 4.1.1, Section 4.1.2 and Section 4.1.3.

The responses of the two models were compared under irradiance variations, load disturbances, and three-phase short-circuit faults.

### 4.1. Assessment of Simulation Accuracy

The reconstruction accuracy of the MFDR model is evaluated using the root-mean-square error (RMSE). Both models used the same simulation time base, and their output signals were recorded at the same sampling instants. Therefore, the two datasets shared the same time vector and were compared directly according to their common timestamps. No resampling, interpolation, or additional time-shift correction was required. For each reported steady-state or transient RMSE, the same evaluation interval was selected from both datasets before error calculation.

(32) $$\mathrm{RMSE}=\sqrt{\frac{1}{N}\sum_{n=1}^{N}{\left[y_{\mathrm{MFDR}}(n)-y_{\mathrm{trad}}(n)\right]}^{2}}$$

where *N* is the number of sampled points within the selected evaluation interval, and *y*_MFDR_ (*n*) and *y*_trad_ (*n*) are the responses of the MFDR model and the traditional model, respectively, at the *n*-th common sampling instant.

The switching-based traditional model is used as the common numerical benchmark because it allows the combined effects of the PV-side, battery-converter, and grid-inverter reconstructions to be evaluated under identical system and computational conditions. Previous studies have already reported method-specific comparisons between representative reduced-order inverter models and detailed EMT models [15,16]. The present comparison therefore focuses on the integrated accuracy and computational performance of the complete MFDR framework.

#### 4.1.1. Accuracy Assessment Under Irradiance Variations

To evaluate the dynamic accuracy of the MFDR model under environmental variations, the irradiance increases from 800 to 1000 W/m^2^ at 0.6 s, rises to 1200 W/m^2^ at 1.2 s, and returns to 800 W/m^2^ at 1.8 s.

**Figure 12:**
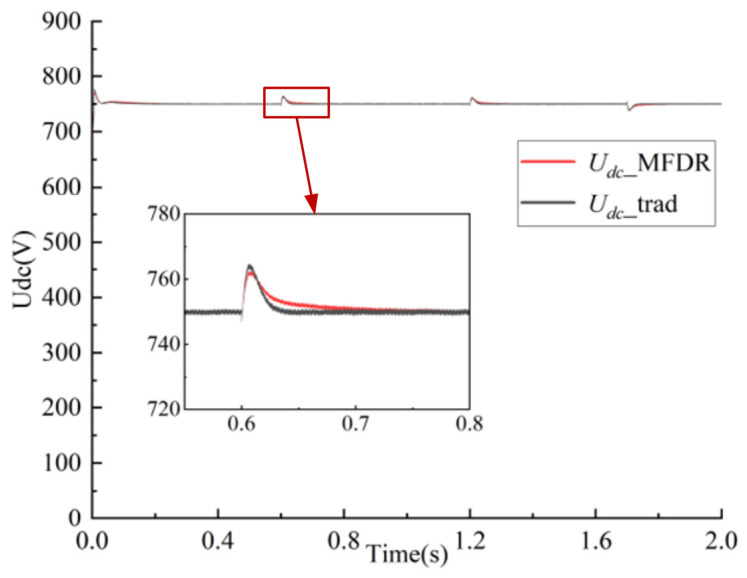
Comparison of DC-bus voltage dynamic response under irradiance-step disturbances.

Figure 12 shows the comparison of the DC-bus voltage response between the MFDR model and the traditional model under fluctuating environmental conditions.

As shown in Figure 12, the traditional model exhibits a switching-frequency voltage ripple during steady-state operation, corresponding to 0.33% of the 750 V DC-bus voltage. The MFDR represents the averaged voltage trajectory and does not reproduce the switching pulses. The steady-state RMSE between the two models is 0.15 V, equivalent to only 0.02% of the reference voltage.

At 0.6 s, the irradiance increase produces a temporary surplus of PV power. Before the inverter and battery controllers rebalance the power flow, the excess energy charges the DC-link capacitor and raises the bus voltage. The traditional model reaches 765 V and returns to the steady-state range within 0.08 s. The MFDR model predicts a peak of 764 V, resulting in an absolute peak error of 1 V and a relative peak error of 0.13%. The transient RMSE is 0.42 V. The results show that the MFDR model retains the capacitor energy accumulation and controller-regulated voltage recovery associated with the irradiance disturbance. The smoother MFDR waveform results from the omission of switching dynamics rather than an error in the reconstructed DC-bus voltage response. It shows that the averaging of the switching effects does not produce a noticeable deviation in the steady-state voltage.

**Figure 13:**
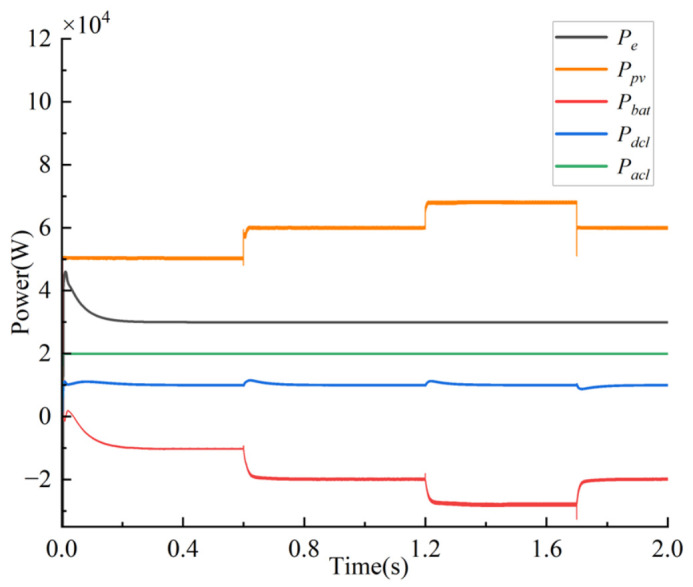
Power redistribution obtained from the MFDR model under irradiance variations.

Figure 13 further presents the power redistribution obtained from the MFDR model under irradiance variations. It is used to explain the power-balance mechanism underlying the DC-bus voltage response shown in Figure 12. *P_e_*, *P_pv_*, *P_bat_*, *P_dcl_*, and *P_acl_* denote the grid-side power demand, PV output power, battery power, DC-load power, and AC-load power, respectively.

At 1.2 s, *P_pv_* increases from 60.0 kW to approximately 68.5 kW. Meanwhile, *P_bat_* changes from approximately −20.0 kW to −28.5 kW. Since negative battery power denotes charging, the battery absorbs an additional 8.5 kW, which is equal to the increase in PV output. Consequently, the combined power of *P_pv_* and *P_bat_* remains approximately 40 kW. The DC and AC load powers are maintained at approximately 10 kW and 20 kW, respectively, while the grid-side demand remains nearly unchanged after the initial transient.

This power redistribution explains the voltage recovery shown in Figure 12. After a brief charging interval of the DC-link capacitor, most of the additional PV power is absorbed by the battery. The energy stored in the DC-link capacitor therefore stops increasing, and the bus voltage returns to its reference range. When the irradiance decreases, the battery charging power decreases and compensates for the reduction in PV output. The MFDR model reproduces this balanced power redistribution without introducing a sustained mismatch among the PV unit, battery, loads, and grid.

#### 4.1.2. Accuracy Assessment Under an Active-Load Disturbance

**Figure 14:**
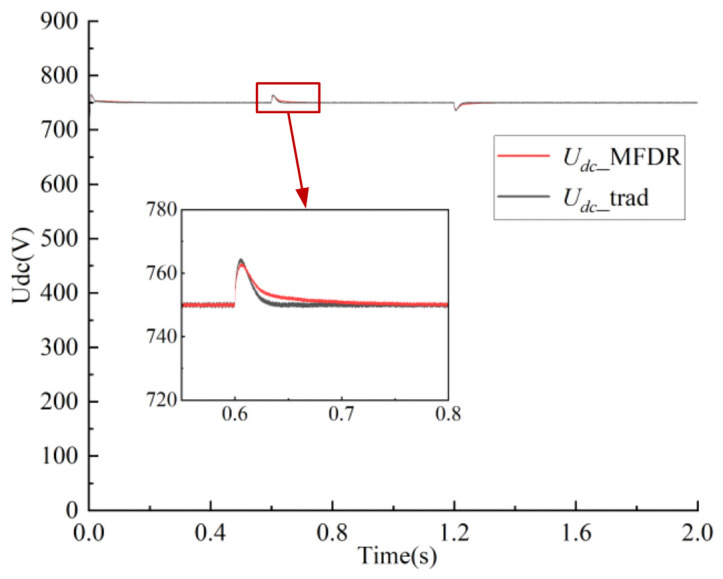
Comparison of DC-bus voltage dynamic response under active-load step.

In this section, the PV output is maintained at 60 kW, while the active-load demand is set to 30 kW. At 0.6 s, the load decreases to 20 kW and returns to 30 kW at 1.2 s. Figure 14 compares the DC-bus voltage responses of the MFDR and traditional models.

When the load decreases by 10 kW at 0.6 s, the unchanged PV generation creates a temporary power surplus. This surplus charges the DC-link capacitor, causing the bus voltage to rise. The traditional model reaches a peak of 764.5 V, corresponding to a 14.5 V increase and an overshoot percentage of 1.93% relative to the 750 V reference. The voltage returns to the steady-state range within 0.12 s as the inverter and battery controllers redistribute the surplus power.

The MFDR model reaches a peak voltage of 762.8 V, which is 1.7 V lower than that of the traditional model and corresponds to a relative peak error of 0.22%. The RMSE over the complete interval is 0.86 V, equivalent to 0.115% of the reference voltage. When the load returns to 30 kW at 1.2 s, the additional power demand is supplied temporarily by the DC-link capacitor, causing a short voltage decrease. The subsequent recovery indicates that the MFDR model reproduces the dominant capacitor energy exchange and power rebalancing dynamics under load disturbances.

**Figure 15:**
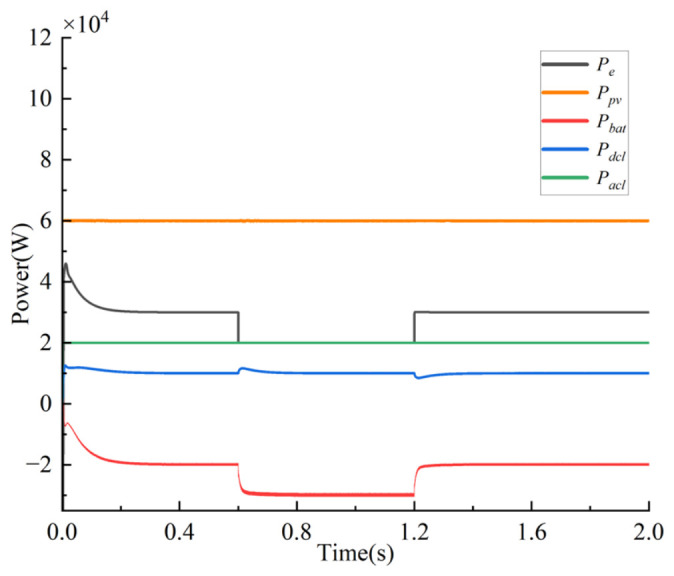
Power redistribution obtained from the MFDR model under an active-load disturbance.

Figure 15 presents the power redistribution obtained from the MFDR model under the active-load disturbance. It is used to explain the power-balance mechanism underlying the DC-bus voltage response shown in Figure 14.

The PV output power remains constant at 60 kW throughout the test. At 0.6 s, the active-load demand decreases by 10 kW. Because the PV generation does not change simultaneously, the system temporarily develops a 10 kW power surplus.

With the convention that negative *P_bat_* denotes battery charging, *P_bat_* decreases from approximately −20 kW to −30 kW. Thus, the battery charging power increases by 10 kW, exactly matching the load reduction. Consequently, the net power supplied by the PV–battery combination decreases from approximately 40 kW to 30 kW, maintaining the system-level power balance.

This 10 kW adjustment explains why the DC-bus voltage in Figure 14 returns rapidly after the initial rise. Once the battery absorbs the surplus power, the DC-link capacitor no longer accumulates energy. When the load returns to 30 kW at 1.2 s, *P_bat_* returns to −20 kW, and the initial power distribution is restored. The MFDR model therefore reproduces both the magnitude and direction of the battery power adjustment required by the load change.

**Figure 16:**
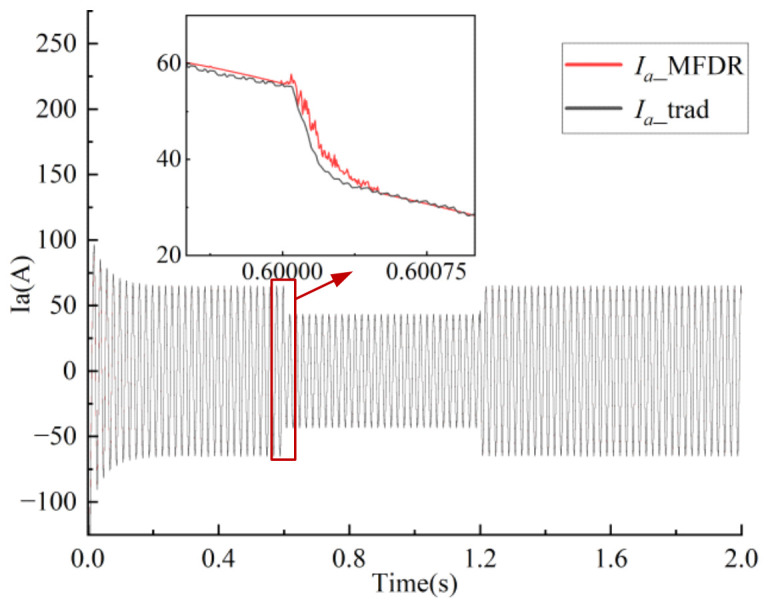
Phase-A current responses at the PCC under an active-load disturbance.

Figure 16 compares the phase-A currents at the point of common coupling (PCC) obtained from the MFDR and traditional models.

Taking the fundamental component of the traditional model current as the reference, the steady-state and transient RMSEs are 0.08 A and 0.37 A, respectively. The transient RMSE corresponds to less than 0.62% of the approximately 60 A current amplitude.

At 0.6 s, the active load decreases by 10 kW. As shown in Figure 15, the battery charging power simultaneously increases by 10 kW, so the power exchanged through the grid-side inverter does not undergo a sustained change. Consequently, the phase-A current maintains approximately the same fundamental amplitude and phase before and after the disturbance. Only a short transition appears while the battery converter and inverter current controller redistribute the power. The maximum normalized instantaneous tracking error during the short transition remains below 2%.

The preservation of the current zero crossings and fundamental trajectory indicates that the MFDR model retains the grid-synchronization and current-control responses required for monitoring. The smoother current trajectory results from the averaging of the switching effects in the reconstruction process.

#### 4.1.3. Accuracy Assessment Under Three-Phase Short-Circuit Fault

A three-phase short-circuit fault is applied at the PCC at 0.6 s and cleared at 0.8 s, giving a fault duration of 0.2 s.

**Figure 17:**
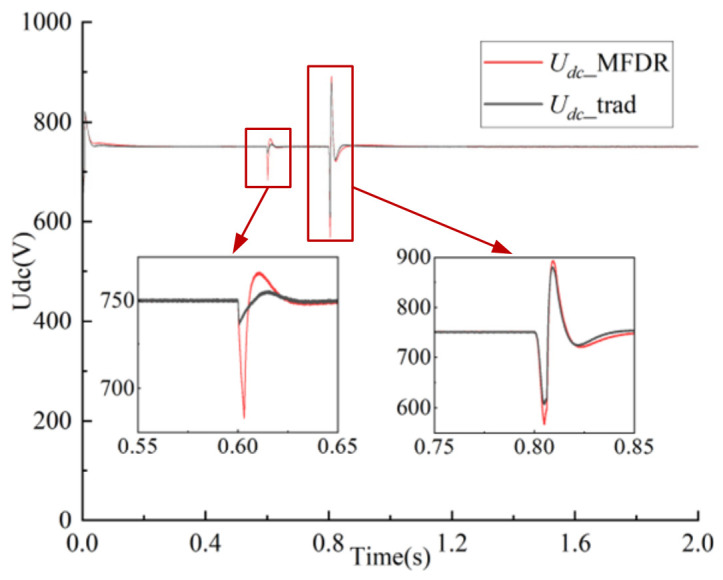
DC-bus voltage responses during a three-phase short-circuit fault.

Figure 17 compares the DC-bus voltage responses throughout the fault cycle. At fault inception, the collapse of the PCC voltage abruptly changes the active-power transfer capability of the grid-side inverter.

The PCC-voltage collapse reduces the active-power transfer capability of the grid-connected inverter and creates a temporary imbalance between the DC and AC sides. During the fault interval, the coordinated inverter and battery controls restore the DC-bus voltage to approximately 750 V. The RMSE between the MFDR and traditional-model voltage trajectories is 1.24 V, corresponding to 0.165% of the reference voltage.

The largest voltage deviation occurs when the fault is cleared at 0.8 s. At this instant, the rapid restoration of the PCC voltage is accompanied by PLL resynchronization, current-controller adjustment, and the re-establishment of inverter active-power transfer. These coupled responses produce a faster change in AC-side power than the irradiance and load disturbances, during which grid synchronization is maintained.

The rapid recovery of inverter power creates a short-term mismatch between the DC-side input power and the AC-side output power, which is absorbed by the DC-link capacitor. Therefore, a small timing or amplitude difference in the inverter response can produce a larger deviation in the DC-bus voltage extrema. The traditional model reaches a minimum of approximately 600 V, whereas the MFDR model predicts 585 V, corresponding to an error of 15 V or 2.5%. The subsequent recovery peak reaches 892 V in the MFDR model, with a relative error of approximately 1.3%. Despite these localized deviations, the two models show consistent voltage-depression, recovery, and rebound trends.

**Figure 18:**
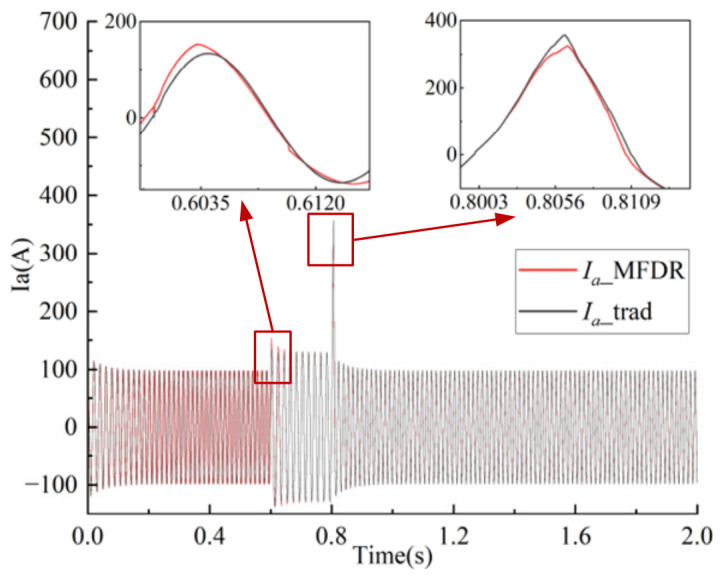
Phase-A current waveforms at PCC under a three-phase short-circuit fault.

Figure 18 compares the phase-A currents at the PCC during the three-phase short-circuit fault.

At 0.6 s, the abrupt collapse of the PCC voltage produces a large voltage difference across the grid-side filter inductance. Because the current controller cannot respond instantaneously, the resulting *di*/*dt* generates a short-duration current impulse exceeding 200 A. After this initial impulse, the current controller enters the limiting region and constrains the converter current to approximately 1.2 times its rated value, thereby preventing a sustained overcurrent.

After the fault is cleared, the PCC voltage and inverter terminal-voltage reference recover rapidly. A second current transient occurs while the PLL re-establishes phase synchronization and the current controller adjusts the inverter current. Because these processes occur within a short interval, small differences in control-response timing produce larger local phase and amplitude deviations than those observed under irradiance and load disturbances. Nevertheless, the normalized phase- and amplitude-tracking errors remain below 2% and 3%, respectively, and the RMSE over the complete fault and recovery periods is 0.62 A. The results indicate that the MFDR model retains the current-limiting behavior and the overall post-fault recovery trajectory required for system-level LVRT monitoring. For applications requiring accurate first-cycle current peaks, rapid synchronization details, or protection-threshold assessment, a switching-level EMT model remains preferable.

**Figure 19:**
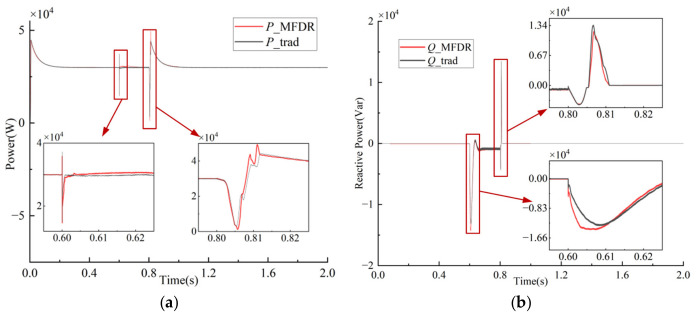
System power responses during a three-phase short-circuit fault. (**a**) Active power response. (**b**) Reactive power response.

Figure 19 compares the active and reactive power responses of the MFDR and traditional models during the three-phase short-circuit fault.

As shown in Figure 19a, during the fault interval, the active power decreases from 30 kW to approximately 0 kW within 5 ms because the PCC-voltage collapse restricts power transfer through the inverter. The RMSE between the MFDR and averaged traditional-model power responses is 0.18 kW, corresponding to 0.60% of the pre-fault power.

When the fault is cleared, the recovered PCC voltage restores the active-power transfer path. At the same time, energy stored in the DC-link capacitor is released through the inverter, producing a short power peak. The traditional and MFDR models reach 48.2 kW and 47.8 kW, respectively. The peak error is 0.4 kW, or 0.83%. The MFDR model therefore retains both the active-power reduction during the fault and the energy-release process immediately after fault clearing.

Figure 19b shows that the reactive-current command produces a negative reactive-power transient of −16.6 kvar at fault inception. Following fault clearing, the rapid recovery of the PCC voltage, together with the transient adjustment of the PLL and current controller, produces a positive reactive-power peak of 13.4 kvar. The MFDR model reproduces both extrema with relative errors below 2%, and the overall reactive-power RMSE is 0.22 kvar.

Together, the active- and reactive-power results show that the MFDR model retains the active-power reduction, reactive-current support, and post-fault power recovery required for low-voltage ride-through assessment.

A comparison among the three disturbance cases shows that the reconstruction error increases with the response rate. The irradiance variation is dominated by the relatively slow redistribution of power among the PV unit, DC-link capacitor, and battery, resulting in small steady-state and transient voltage errors. The active-load step additionally excites the coupled DC-link, battery-converter, and grid-side current-control dynamics, leading to a moderately larger voltage deviation. During the short-circuit fault, the main differences are concentrated around fault inception and clearing, where the voltage and current change most rapidly. The absence of sustained deviations during the subsequent recovery indicates that the dominant energy exchange, current response, and post-fault restoration processes are retained, while the remaining errors are mainly associated with the fastest transient components.

### 4.2. Computational Load and Scalability Assessment

To evaluate the computational efficiency and scalability of the proposed MFDR model, CPU utilization and maximum step execution time are used. CPU utilization represents the processor resources required during continuous operation, while the maximum step execution time indicates whether each calculation can be completed within the prescribed real-time simulation step. The test systems are progressively extended from a microgrid to the IEEE 33-bus system, the IEEE 118-bus system, and an extended MFDR model containing 1069 total states. To characterize the network scale of the benchmark cases, the microgrid, IEEE 33-bus system, and IEEE 118-bus system involve 27, 99, and 354 phase-domain network variables, respectively. These values represent the network-state dimensions and exclude the internal states of the converters and controllers. For each comparative case, the traditional and MFDR models use the same network topology and therefore have the same network-state dimension.

Table 2 compares the computational performance of traditional and MFDR models in the microgrid and IEEE 33-bus system under single-core execution.

For the microgrid, CPU utilization decreases from 37.9% to 16.7%, corresponding to a reduction of 55.9%. The maximum step execution time decreases from 23 μs to 11 μs, representing a reduction of 52.2%.

For the IEEE 33-bus system, CPU utilization decreases from 84.4% to 28.2%, corresponding to a reduction of 66.6%. The maximum step execution time decreases from 26 μs to 16 μs, representing a reduction of 38.5%. Both systems complete each calculation within the fixed 50 μs simulation step.

The MFDR model replaces switching-event calculations with reconstructed state equations, thereby reducing the switching-state updates and topology-dependent operations required within each simulation step. The larger CPU-utilization reduction in the IEEE 33-bus system indicates that this computational benefit becomes more evident as additional converter units and network components are included.

**Figure 20:**
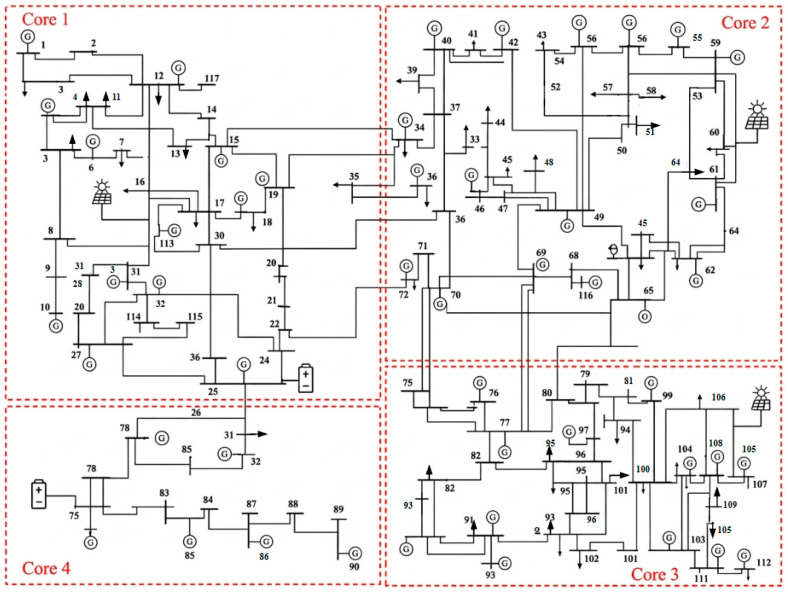
Multi-core partitioning of the IEEE 118-bus system with distributed resources.

To further evaluate the computational performance under a large network, the IEEE 118-bus system was deployed on the MT8020 real-time simulator using partitioned multi-core execution. The partitioning structure is shown in Figure 20.

The IEEE 118-bus system is divided into four computational regions according to network connectivity and workload distribution. Boundary variables are exchanged between adjacent cores at each simulation step to preserve the electrical coupling among the partitioned regions. This reduces the computational burden on a single processor. The traditional model and the MFDR model use the same network partition, core allocation, operating condition, and simulation step.

Table 3 compares the computational performance of the traditional model and MFDR model in the IEEE 118-bus system under multi-core execution.

The CPU utilization of the traditional model ranges from 67.4% to 73.3%, with an average value of 70.43%. After applying the MFDR method, the corresponding range decreases to 38.4−43.9%. The average CPU utilization is reduced to 41.08%, representing a reduction of 41.7%.

The reduction is consistent across all four cores. The single-core CPU utilization decreases by 40.1–43.0%. Moreover, the difference between the maximum and minimum core utilization remains small. No obvious single-core bottleneck is observed.

The traditional model requires maximum step execution times of 38 μs among the four cores. In comparison, the MFDR model requires only 27 μs. The average value decreases from 36.0 μs to 26.3 μs, corresponding to a reduction of 26.9%. The overall maximum step execution time is also reduced from 38 μs to 27 μs, with a reduction of 28.9%.

Under the fixed simulation step of 50 μs, the maximum step execution times of both models remain below the prescribed limit. However, the MFDR model completes each simulation step in a shorter time. It therefore imposes less computational pressure on the real-time simulator and is more suitable for further system expansion and the integration of additional converter devices.

**Figure 21:**
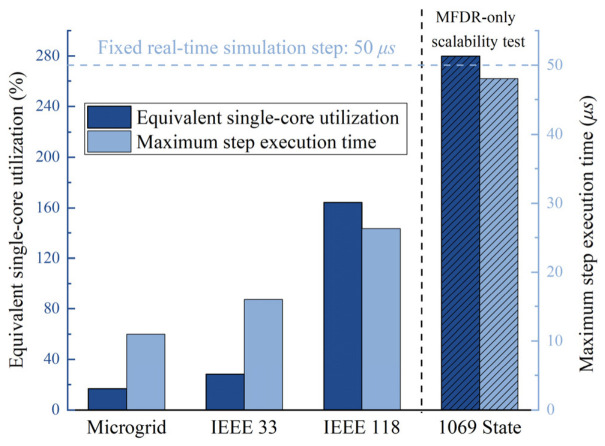
Computational scalability of the MFDR model.

To compare cases executed with different numbers of CPU cores, an equivalent single-core load *L_eq_* is defined as a unified aggregate indicator:

(33) $$L_{eq}=\sum_{i=1}^{N_{c}}U_{i}$$

where *U_i_* is the CPU utilization of the *i*-th active computational core and *N_c_* is the number of active cores. For a single-core case, *L_eq_* is equal to the measured CPU utilization. For a multi-core case, it represents the sum of the utilizations of all active cores. The equivalent single-core load is adopted in Figure 21 as a unified indicator.

As shown in Figure 21 below, both the equivalent single-core utilization and the maximum step execution time increase with system scale. The 1069-state case is included as an MFDR-only scalability stress test and does not involve a reduction comparison with the traditional model. The equivalent single-core utilization values for the microgrid and IEEE 33-bus systems are 16.7% and 28.2%, respectively. Their corresponding maximum step execution times are 11 μs and 16 μs.

These results indicate a relatively low computational burden for the small-scale systems.

When the system is expanded to the IEEE 118-bus case, the *L_eq_* increases to 164.32%, and the maximum step size rises to 27 μs. This means that the model requires computational resources equivalent to approximately 1.64 fully occupied CPU cores. However, the maximum step execution time remains well below the fixed real-time simulation step of 50 μs, showing that the MFDR model can still support real-time simulation for a large-scale power network.

Under the four-core execution configuration, the extended model with 1069 state variables reaches an equivalent single-core load of 279.8% and a maximum step execution time of 48 μs. This load is equivalent to approximately 2.8 fully utilized CPU cores. The calculation can still be completed within the prescribed 50 μs simulation step, but the execution time is already close to the real-time limit of the current platform.

Nevertheless, all tested MFDR cases complete each calculation within the fixed 50 μs simulation step.

The computational indices describe two different aspects of real-time performance. CPU utilization characterizes the average processor demand during continuous execution, whereas the maximum step execution time determines whether each calculation can be completed within the prescribed simulation step. In the four-core IEEE 118-bus case, the reduction in average per-core CPU utilization from 70.43% to 41.08% indicates a substantial decrease in the sustained computational load. Meanwhile, the decrease in the maximum step execution time from 38 μs to 27 μs increases the available real-time execution margin. Because the traditional and MFDR models use the same network topology, partition, and network-state dimension, these reductions mainly arise from replacing switching-dependent calculations with reconstructed state and frequency-domain equations rather than from reducing the network scale. As the system is extended, both the equivalent single-core load and the maximum step execution time increase. The extended model reaches 48 μs within the fixed 50 μs step, indicating that real-time execution remains feasible, although the small remaining margin also defines the practical limit of the current hardware and partitioning configuration.

## 5. Conclusions

To address the high computational burden of real-time simulation for large-scale PV systems with battery storage, this study developed a Multi-Feature Dynamic Reconstruction (MFDR) method for the real-time grid monitoring of PV systems with battery storage. The proposed method integrates environmental feature mapping for the PV unit, an averaged-state representation of the bidirectional battery converter, and a frequency-domain Norton representation of the grid-connected inverter. Based on the comparative accuracy tests and the computational scalability assessment, the main conclusions are summarized as follows:

(1) The MFDR model captures the principal voltage, current, and power responses of the PV–battery storage system under irradiance variations and load disturbances. During the irradiance-step test, the steady-state and transient RMSEs of the DC-bus voltage are 0.15 V and 0.42 V, respectively, while the relative peak error is 0.13%. During the 10 kW load reduction, the battery charging power increases by 10 kW. The DC-bus voltage RMSE is 0.86 V, and the transient phase-A current RMSE is 0.37 A. These results confirm that the averaged formulation preserves the system-level dynamic behavior required for real-time grid monitoring.
(2) Under the three-phase short-circuit fault, the MFDR model closely matches the traditional model in terms of DC-bus voltage variation, reactive-current support, and post-fault power recovery. The DC-bus voltage RMSE is 1.24 V. The relative errors at the voltage minimum and recovery peak are 2.5% and 1.3%, respectively. The phase-A current RMSE over the fault and recovery periods is 0.62 A, and the amplitude-tracking error during recovery remains below 3%. The active- and reactive-power RMSEs are 0.18 kW and 0.22 kvar. Therefore, the MFDR model preserves the main transient characteristics required for system-level fault analysis and low-voltage ride-through assessment.
(3) The MFDR model reduces both CPU utilization and step execution time. For the microgrid, CPU utilization and maximum step execution time decrease by 55.9% and 52.2%. For the IEEE 33-bus system, the corresponding reductions are 66.6% and 38.5%. In the four-core IEEE 118-bus case, the average per-core CPU utilization decreases from 70.43% to 41.08%, and the maximum step execution time decreases from 38 μs to 27 μs. Under the four-core execution configuration, the model with 1069 state variables has an equivalent single-core load of 279.8% and a maximum step execution time of 48 μs; therefore, each calculation can still be completed within the fixed 50 μs simulation step.

The MFDR method is intended for system-level real-time monitoring of voltage, current, and power states and does not resolve individual switching-device waveforms. The present reconstruction-accuracy assessment uses the traditional switching-based model as the numerical reference, while synchronized field measurements from an operating PV–battery system are not included. Therefore, the reported errors characterize the model-to-model reconstruction consistency under the tested CHIL conditions. Future work will incorporate site-measured voltage, current, and power data under representative operating conditions to evaluate the field-level accuracy and parameter adaptability of the MFDR model. The simulation scale will also be extended by incorporating additional network nodes, PV units, battery storage units, and power-electronic interfaces, and its computational performance will be evaluated under larger multi-core partitions.

## Figures and Tables

**Table 1:** Main parameters and test conditions of the microgrid case.

Parameter	Value
System frequency *f* (Hz)	50
PV rated operating point *V_m_* (V)/*I_m_* (A)	300/200
Rated PV power *P_pv_* (kW)	60
PV open-circuit voltage/short-circuit current *V_oc_* (V)/*I_sc_* (A)	375/220
Energy storage capacity (Ah)	40
DC-link voltage reference *U_dc_* (V)	750
DC-link capacitance *C* (F)	0.01
Filter inductance *L* (H)	0.0005
Resistance *R* (Ω)	0.1
DC-load power *P_dcl_* (kW)	10
AC-load power *P_acl_* (kW)	20
Simulation step (μs)	50

**Table 2:** Computational performance comparison under single-core execution.

Test System	CPU Utilization (%)		Reduction (%)	Maximum Step Execution Time (μs)		Reduction (%)
	Traditional	MFDR		Traditional	MFDR	
Microgrid	37.9	16.7	55.9	23	11	52.2
IEEE 33	84.4	28.2	66.6	26	16	38.5

**Table 3:** Computational performance of the IEEE 118-bus system under four-core execution.

Core Index	CPU Utilization (%)		Reduction (%)	Maximum Step Execution Time (μs)		Reduction (%)
	Traditional	MFDR		Traditional	MFDR	
Core 1	73.3	43.9	40.1	38	27	28.9
Core 2	70.6	41.2	41.6	36	27	25.0
Core 3	70.4	40.8	42.0	36	26	27.8
Core 4	67.4	38.4	43.0	34	25	26.5
Average	70.43	41.08	41.7	36.0	26.3	26.9
Maximum	73.3	43.9	40.1	38	27	28.9

## Data Availability

Data are contained within the article.

## Abbreviations

The following abbreviations are used in this manuscript:

PV	Photovoltaic
MFDR	Multi-Feature Dynamic Reconstruction
PLL	Phase-locked loop
EMT	Electromagnetic transient
STC	Standard Test Conditions
PCC	Point of common coupling
SOC	State of charge
MPPT	Maximum power point tracking
RMSE	Root-mean-square error
CHIL	Controller hardware-in-the-loop
LFRM	Low-frequency reconstruction model
FDDR	Frequency-domain dynamic reconstruction
**Nomenclature**	
*S*	Solar irradiance
*T*	PV-module temperature
*P_m_*	Maximum available PV power
*I_ref_*	Equivalent PV-side current command
*U_dc_*	DC-bus voltage
*i_L_*	Battery-converter inductor current
*d*(*t*)	Averaged duty ratio of the battery converter
Δ*t*	Simulation time step
***i****_abc_*	Three-phase inverter-current vector
***u****_abc_*	Three-phase grid-voltage vector
*ω*_0_	Fundamental angular frequency
*T_s_*	Switching period
*K*	Maximum retained dynamic-phasor order
*Y*_*eq*,*k*_	Equivalent admittance matrix of the *k*-th dynamic-phasor component
*J*_*eq*,*k*_	Equivalent current source of the *k*-th dynamic-phasor component
*P_e_*	Grid-side power demand
*P_pv_*	PV output power
*P_bat_*	Battery power
*P_dcl_*	DC-load power
*P_acl_*	AC-load power
*y*_MFDR_	Response obtained from the MFDR model
*y*_trad_	Response obtained from the traditional model
*L_eq_*	Equivalent single-core CPU utilization
